# Referral patterns and findings of patients undergoing CT coronary angiography

**DOI:** 10.5339/qmj.2026.22

**Published:** 2026-06-04

**Authors:** Omar K. Al-Saaidi, Saleh Baawain, Malik Al Ghafri, Sunil K. Nadar

**Affiliations:** 1 Directorate of Nursing, Sultan Qaboos University Hospital, Muscat, Oman; 2 Department of Radiology, Sultan Qaboos University Hospital, Muscat, Oman; 3 Department of Clinical Physiology, Sultan Qaboos University Hospital, Muscat, Oman; 4 Department of Medicine, Sultan Qaboos University Hospital, Muscat, Oman

**Keywords:** Coronary artery disease, CT coronary angiography, coronary ectasia, coronary abnormalities

## Abstract

**Background:**

Computed tomography coronary angiography (CTCA) is widely used to rule out significant coronary artery disease in patients who present with chest pain, with many international guidelines recommending its use. However, there is a tendency for it to be used indiscriminately.

**Objectives:**

This study aimed to evaluate the requesting patterns of CTCA at our institution and the outcomes of these patients. We also aimed to study the prevalence of coronary anomalies observed in these patients.

**Methodology:**

This was a retrospective study of patients who had undergone a CTCA at Sultan Qaboos University Hospital, Muscat, over 5 years (2019–2023). Information was obtained from the electronic patient records. Data are presented as a number (percentage) or mean ± standard deviation as appropriate.

**Results:**

The CTCAs of 1000 patients (mean age, 51.1 + 13.5 years; 606 or 60.6% male) were analyzed. Chest pain was the main indication for requesting CTCA in 670 patients (67.2%), with breathlessness and palpitations as the other indications in 198 (19.8%) and 110 (11%) patients, respectively. Of the findings, 835 (83.5%) patients had right dominance, 92 (9.2%) had left dominance, and the remainder were co-dominant. Of the congenital abnormalities noted, 25 (2.5%) had abnormal origins (right coronary artery 9, circumflex 9), and 27 had aneurysmal or ectatic vessels. One hundred and fifty-two patients had evidence of >50% stenosis, of which 131 patients (13.1%) subsequently had an angiogram. Of these, 77 (7.7%) had evidence of significant disease and underwent intervention.

**Conclusion:**

CTCA is often requested for indications other than chest pain. The use of CTCA resulted in only a small proportion of patients proceeding for invasive coronary angiography, and helps in triaging patients better.

## 1. INTRODUCTION

Cardiovascular diseases (CVDs) remain an important cause of morbidity and mortality worldwide, resulting in an increased healthcare cost burden. In 2019, approximately 17.9 million deaths were attributed to CVDs, accounting for 32% of all deaths globally.^1^ Eighty-five percent of these deaths were linked to acute cardiovascular events, specifically myocardial infarctions and cerebrovascular strokes.^1^ Identifying early CVDs is important in preventing worsening disease and adverse outcomes. Easy-to-implement, non-invasive tests are increasingly being used to detect early disease. In particular, computed tomography coronary angiography (CTCA) is increasingly being used in the early detection of coronary artery disease.^2^ Newer generation, improved technological advances with newer machines enable the detection and measurement of plaque features, capabilities previously limited to invasive tests.^3^

CTCA is increasingly utilized as an initial diagnostic tool for patients with chest pain, aiding in both diagnosis and management planning.^4^ It is particularly suitable for guiding treatment decisions in patients presenting with stable chest pain and a low to intermediate pre-test likelihood of coronary artery disease. According to the 2024 ESC guidelines for managing chronic coronary syndromes, CTCA is recommended as the preferred imaging modality for patients with a low clinical probability of coronary artery disease (CAD), no prior CAD diagnosis, and features that are likely to result in high-quality imaging.^5^ Similarly, CTCA offers exceptional spatial and temporal resolution, 3D visualization, and a broad field of view, enabling detailed assessment of coronary abnormalities such as their origin, course, ectasia, and aneurysms.^6^

With the increased availability and access to CTCA as a non-invasive method of assessing coronary arteries, there could be a tendency for it to be used indiscriminately. This is especially important in publicly funded, resource-limited health care systems. The aim of this study is therefore two-fold. First, we aim to study the requested patterns for CTCA at our institution in terms of symptoms. Second, we aim to assess the prevalence, anatomical distribution, and clinical outcomes of coronary artery stenosis and abnormalities detected through CTCA at a single tertiary center in Oman.

## 2. METHODOLOGY

This was a retrospective study involving the medical records of patients referred for CTCA at a tertiary center in Oman. We examined all the records of eligible patients who were referred to for CTCA from January 1, 2019, to December 31, 2023.

This study commenced after obtaining ethical approval from the Medical Research Ethics Committee (MREC number: 2455; date: June 7, 2021) of Sultan Qaboos University Hospital, Muscat, Oman. The list of patients who had undergone CTCA was obtained from the Radiology Department of our institution. All patients who underwent CTCA during the study period were eligible for inclusion in the study. We excluded patients where complete clinical information was not available and where the CT scan results were not complete. Patients were excluded if they had previously undergone coronary artery bypass grafting (CABG) or coronary stenting. Cases were also excluded if only CT calcium scoring was performed or if the cardiac CT scans lacked sufficient detail or were considered non-diagnostic due to significant artefacts.

The electronic case records of eligible patients were then reviewed to obtain details such as cardiovascular risk factors, including hypertension, dyslipidemia, diabetes, smoking, familial hypercholesterolemia, asthma, family history of heart disease, patients’ symptoms such as chest pain, breathlessness, or palpitations, and the indication for the study. The CT report was then reviewed, and the relevant information was obtained.

The study was conducted in accordance with the Declaration of Helsinki.

Coronary CT angiography (CTA) was performed using a third-generation dual-source 256-slice CT scanner (SOMATOM Force; Siemens Healthineers, Munich, Germany) with retrospective ECG-gated, low-pitch acquisition (pitch 0.15). Scan parameters included 80 kVp, tube current up to 867 mA with automated modulation, 192 × 0.6 mm collimation, and a 0.25-s gantry rotation time. The mean radiation dose for the coronary CTA phase was CT dose index (CTDI_vol_) 42.86 mGy and dose length product (DLP) 611.58 mGy·cm. Images were reconstructed with a 0.75-mm slice thickness at 65% of the R-R interval, with additional phases from 10% to 90% reconstructed. All data sets were reviewed on a dedicated cardiac analysis workstation (syngo.via; Siemens Healthineers).

Coronary dominance was determined according to standard protocol. If the posterior descending artery (PDA) and the posteroslateral artery (PLA) arise from the right coronary artery (RCA), the circulation is deemed to be “right dominant.” If the PDA and PLA arise from the circumflex artery, then that is classed as “left dominant.” If, however, either the PLA or PDA arose from the RCA and the other from the circumflex, then this was a co-dominant circulation.

The degree of coronary stenosis was categorized into four levels: normal, <50%, >50%, or 100% occlusion. Further specific findings were documented, such as coronary artery anomalies, abnormal origin and course, aneurysms, or ectasia. We checked the records to ascertain whether the patient had undergone invasive coronary angiography. If angiography was performed, the findings were compared with cardiac CT results to evaluate concordance and to determine the need for further interventions.

Statistical analysis was performed using SPSS, version 21.0 (IBM Corp., Chicago, IL). Data are presented as a number (percentage) or mean ± standard deviation if categorical or continuous, respectively.

## 3. RESULTS

A total of 1231 patients had undergone CTCA during the study period. Of these 38 patients had previous CABG, and 188 only had coronary calcium scoring and not a full study. Five patients had no clinical data as they were referred from elsewhere for the scan, and we had no clinical information on our system, and hence were not included in the study. The remaining 1000 CT scans were of good quality and had a full study that was interpretable. All these patients had a full data set. The CT scans of 1000 consecutive patients (mean age, 51.1 + 13.5 years; 606 male, 394 female) were reviewed. The demographics and baseline characteristics are given in Table 1. The main indication for the scan was chest pain (67%), followed by breathlessness (19.8%) and palpitations (11%).

Most of the patients had a right dominant system (835 or 83.5%), followed by left dominant (92 or 9.2%) and co-dominant systems (72 or 7.2%). The summary of the findings is given in Table 2. The majority of the patients had normal scans (836 or 83.6%), with 164 being abnormal. There was a 2.6% prevalence of coronary ectasia, with 13 of these in the RCA. Abnormal course was seen in 2.5% of patients with the RCA (0.9%) and circumflex (0.9%) being the most common. The left main had an abnormal origin in 0.5% of the patients, with the left anterior descending (LAD) being abnormal in 0.2%.

Luminal stenosis of >50% were found in only 8 of the 110 (7.2%) who had presented with palpitations, 113 of the 670 (16.8%) with chest pain, and 31 of the 198(15.6%) with breathlessness.

A total of 131 patients went on to have invasive coronary angiography. This included 86 patients who had >50% stenosis on CTCA and 45 who had normal CTCA. The interval between both tests ranged from 0 to 100 days, with a mean of 28.7 ± 24.2 days and a median of 21.5 days. The angiogram was reported as normal in 54 patients. Of the 45 who had a normal CTCA, 10 had significant stenosis noted in their coronary angiogram. Of the 86 who had abnormal CTCA, 19 were normal, with the remaining demonstrating significant stenosis. This demonstrated a sensitivity of 87%, specificity of 64.8%, positive predictive value of 77.7%, and a negative predictive value of 77.9%

## 4. DISCUSSION

CTCA is an important tool in the diagnostic armamentarium of practicing cardiologists. Although the main indication is to rule out coronary artery disease in low to intermediate-risk patients, it also gives us important information regarding the coronary anatomy and the presence of congenital anomalies and other anatomic aberrancies. In our retrospective study, we found that although the main indication for the test was chest pain, it was also being used for other presenting complaints, where ischemia could be a potential cause of those symptoms, such as ischemic arrhythmias or breathlessness (atypical angina). For patients where the main indication was documented as “palpitations,” it is possible that the clinician wanted to rule out coronary disease as a cause of potential arrhythmias such as ventricular tachycardia. However, this was not clear from the clinical notes.

Alderazi et al. performed a similar audit on their referral patterns for CTCA.^7^ In their study, the authors studied the referral indication in 234 patients. Of these, 75.2% were to rule out CAD, while the others were to rule out cardiac abnormalities, assess post-revascularization, and, as a result of other tests. This is similar to our study, where chest pain was the indication in around two-thirds of patients. In their study, they found that nearly two-thirds of tests were deemed appropriate. In our study, we did not assess appropriateness.

In our cohort of patients, the majority had a right-dominant system, followed by left dominance and co-dominance, which is consistent with previously reported studies.^8,9^ The prevalence of coronary artery ectasia (CAE) was 2.6% in our cohort of patients. Giannoglou et al. also found a similar prevalence of around 2.7% in their cohort of 10,524 patients in their study from Northern Greece.^10^ In their study from Spain, Pinar Bermudez et al. found a slightly higher prevalence of CAE of 3.39% among a cohort of 4332 patients.^11^ On the other hand, Willner et al. found a much lower prevalence of around 0.85% in their cohort.^12^ As with the other studies, the RCA was responsible for 50% of all the ectatic vessels, slightly higher than the 44.21% reported by Giannoglou et al.^10^ and similar to the study by Pinar Bermudez et al. where 49.7% of the cases involved a single vessel, and most frequently the RCA (89.8% of all single vessel ectasia).^11^ There is, however, no clear explanation why the RCA is the most commonly affected artery. Risk factors for CAE appear to be male sex, younger age, and the absence of diabetes.^9^

A previous study from Oman by Al-Umairi et al. reported a lower prevalence of congenital anomalies (<1%) than in our study.^13^ Other studies, such as those by Kashyap et al.^14^ and Szymczyk et al.^15^ also demonstrated comparable results. Our study, however, found a lower prevalence of significant coronary artery stenosis compared to Kralev et al. who reported a 34% incidence of CAD in patients with atrial fibrillation, increasing to 41% in those over 70 years old, with 21% requiring percutaneous coronary intervention (PCI) or CABG.^16^ In contrast, our study found much lower numbers of patients with significant stenosis in the whole group and especially those with indications other than chest pain. One reason for this may be that, in our study, only very low-risk patients underwent CTCA, whereas in the other study, chest pain was not defined.

Wallis et al. emphasized the role of coronary CT in ruling out CAD in low- to intermediate-risk patients, highlighting its high sensitivity 98% and negative predictive value 99%.^17^ Our study further evaluated the accuracy of CTCA, showing a sensitivity of 87%, specificity of 64.8%, positive predictive value of 77.7%, and negative predictive value of 77.9%, indicating that while CTCA is highly sensitive in detecting CAD, it may overestimate stenosis, leading to false positives. Overall, these studies reinforce the importance of risk-based assessment and careful interpretation of coronary CT results to avoid unnecessary invasive procedures.

One of the main limitations of this study is its retrospective design, which relies on existing medical records, potentially leading to missing or incomplete data. Additionally, the study was conducted at a single center, limiting its generalizability to broader populations with different demographic and clinical characteristics. The reliance on CTCA as the primary imaging modality also introduces potential bias, as its moderate specificity (64.8%) may lead to overestimation of stenosis severity, resulting in false positives and unnecessary follow-up procedures. In our center, we do not offer quantitative plaque analysis, which would have an effect on assessing the severity of the lesion. We also do not report on image quality using a Likert scoring system, which might limit the reproducibility of the results. Despite these limitations, our study was conducted on 1000 individuals, which gives validity to our results.

## 5. CONCLUSION

This study provides valuable insights into the prevalence and anatomical distribution of coronary artery stenosis and anomalies in an Omani population using CTCA, which are similar to those reported in the literature. Although CTCA is a valuable tool in the diagnosis of chest pain, it is being used for other indications as well, thus giving it a low positive yield. Optimizing patient selection through adherence to guideline-based pathways could enhance resource use, reduce unnecessary investigations, and ultimately improve patient care.

## ACKNOWLEDGEMENTS

The author(s) have no acknowledgements to declare.

## FUNDING

The author(s) received no financial support for the research, authorship, and/or publication of this article.

## CONFLICT OF INTEREST

The author(s) declare that there is no conflict of interest.

## ETHICAL APPROVAL

The study was approved by the Medical Research Ethics Committee (MREC number: 2455; date: June 7, 2021).

## AUTHOR CONTRIBUTIONS

OKA and MAG collected the data. SB and SKN conceived the project. SKN analyzed the data. OKA, MAG, SB, and SKN all contributed to writing the manuscript. Author(s) testify that all persons designated as authors qualify for authorship and have checked the article for plagiarism. All authors fulfill the criteria for authorship.

## DISCLOSURE OF AI USE

Artificial intelligence was not used in writing this manuscript.

## Figures and Tables

**Table 1.** Baseline characteristics of patients.

Characteristics	Number (*N* = 1000)
Age	51.1 + 13.5 years
Sex (male: female)	606:394 (60.6%:39.4%)
Presentation	
Chest pain	670 (67%)
Breathlessness	198 (19.8%)
Palpitations	110 (11%)
Others	22(2.2%)
Risk factors	
Diabetes	236(23.6%)
Hypertension	343 (34.3%)
Hyperlipidemia	202(20.2%)
Smoker	69(6.9%)
Familial hypercholesterolemia	84(8.4%)

Figures are numbers (%).

**Table 2.** Summary of findings of the CT scans.

	Left main stem (*n* = 1000)	Left anterior descending (*n* = 1000)	Circumflex artery (*n* = 1000)	Right coronary artery (*n* = 1000)
Normal	916 (91.6%)	699 (69.9%)	813 (81.3%)	797(79.9%)
<50% stenosis	76(7.6%)	195 (19.5%)	134(13.4%)	137(13.7%)
>50% stenosis	2 (2%)	92(9.2%)	36(3.6%)	38(3.8%)
100% stenosis	0	5 (0.5%)	2(0.2%)	6(0.6%)
Ectasia/aneurysms	1(0.1%)	7(0.7%)	6(0.6%)	13(1.3%)
Abnormal origin	5(0.5%)	2(0.2%)	9(0.9%)	9(0.9%)

Figures are numbers (%).
